# A common approach for absolute quantification of short chain CoA thioesters in prokaryotic and eukaryotic microbes

**DOI:** 10.1186/s12934-020-01413-1

**Published:** 2020-08-10

**Authors:** Lars Gläser, Martin Kuhl, Sofija Jovanovic, Michel Fritz, Bastian Vögeli, Tobias J. Erb, Judith Becker, Christoph Wittmann

**Affiliations:** 1 Institute of Systems Biotechnology, Saarland University, Saarbrücken, Germany; 2 Max Planck Institute for Terrestrial Microbiology, Marburg, Germany

**Keywords:** *Corynebacterium glutamicum*, *Streptomyces albus*, *Pseudomonas putida*, *Yarrowia lipolytica*, Lysine, Pamamycin, CoA thioester, LC–MS

## Abstract

**Background:**

Thioesters of coenzyme A participate in 5% of all enzymatic reactions. In microbial cell factories, they function as building blocks for products of recognized commercial value, including natural products such as polyketides, polyunsaturated fatty acids, biofuels, and biopolymers. A core spectrum of approximately 5–10 short chain thioesters is present in many microbes, as inferred from their genomic repertoire. The relevance of these metabolites explains the high interest to trace and quantify them in microbial cells.

**Results:**

Here, we describe a common workflow for extraction and absolute quantification of short chain CoA thioesters in different gram-positive and gram-negative bacteria and eukaryotic yeast, i.e. *Corynebacterium glutamicum*, *Streptomyces albus*, *Pseudomonas putida*, and *Yarrowia lipolytica*. The approach assessed intracellular CoA thioesters down to the picomolar level and exhibited high precision and reproducibility for all microbes, as shown by principal component analysis. Furthermore, it provided interesting insights into microbial CoA metabolism. A succinyl-CoA synthase defective mutant of *C. glutamicum* exhibited an unaffected level of succinyl-CoA that indicated a complete compensation by the l-lysine pathway to bypass the disrupted TCA cycle. Methylmalonyl-CoA, an important building block of high-value polyketides, was identified as dominant CoA thioester in the actinomycete *S*. *albus*. The microbe revealed a more than 10,000-fold difference in the abundance of intracellular CoA thioesters. A recombinant strain of *S. albus*, which produced different derivatives of the antituberculosis polyketide pamamycin, revealed a significant depletion of CoA thioesters of the ethylmalonyl CoA pathway, influencing product level and spectrum.

**Conclusions:**

The high relevance of short chain CoA thioesters to synthetize industrial products and the interesting insights gained from the examples shown in this work, suggest analyzing these metabolites in microbial cell factories more routinely than done so far. Due to its broad application range, the developed approach appears useful to be applied this purpose. Hereby, the possibility to use one single protocol promises to facilitate automatized efforts, which rely on standardized workflows.

## Background

Microbial cell factories are a key to the bio-based industry [1]. Upgrading and streamlining their biocatalytic activity through systems metabolic engineering requires detailed understanding of the underlying metabolism [2–5]. Among other techniques, the assessment of intracellular metabolite levels and pathway fluxes has proven valuable to understand metabolic network function and its regulation and derive novel targets for strain engineering [1, 6].

A relevant group of metabolites are thioesters, esters between a carboxylic acid and a thiol. In microbial metabolism, the best-known and most relevant thioesters are short chain CoA thioesters, derivatives of coenzyme A (CoA) [7, 8]. Notably, CoA thioesters such as acetyl-CoA and succinyl-CoA participate in 5% of all enzymatic reactions and at least one-third of all cellular carbon is typically metabolized through a CoA thioester [7]. As example, they provide activated groups to drive the anabolic synthesis of cellular constituents such as peptides, fatty acids, sterols, and terpenes, display intermediates of catabolic pathways, and are essential to central energy metabolism [8]. Today, bioinformatics databases reveal more than two hundred naturally occurring CoA thioester derivatives [8], of which a core spectrum between approximately 5–10 compounds is potentially present in most microbes, based on their genomic repertoire [9, 10].

From a commercial perspective, CoA thioesters display building blocks of a wide range of industrially interesting products. Prominent examples are polyketides [11], polyunsaturated fatty acids (PUFAs) [12], polyhydroxyalkanoates (PHAs) [13], biofuels [14], amino acids [15], and dicarboxylic acids [16], among others [17]. This relevance might explain the increasing interest to trace CoA thioesters. Previous efforts have provided different experimental approaches, each specifically designed for a particular microbe, including indirect analysis of CoA thioesters via measurement of the respective organic acid, isotope dilution and enzymatic assays [17–20].

In this work, we have set up a sensitive, robust, and reproducible workflow to quantify short chain CoA thioesters in microbes. For this purpose, we adapted a previous protocol, used to assess a wide spectrum of CoA thioesters in the methylotrophic bacterium *Methylobacterium extorquens* [21]. After improvement and careful validation, we demonstrated the approach for industrially relevant microorganisms, which utilize CoA thioesters to form value-added products: the gram-positive bacteria *Corynebacterium glutamicum* [15], and *Streptomyces albus* [11], the gram-negative bacterium *Pseudomonas putida* [13], and the eukaryotic yeast *Yarrowia lipolytica* [12].

## Results

### Set up and validation of a single protocol for extraction and quantification of short chain CoA thioesters in gram-positive and gram-negative bacteria and eukaryotic yeast

A synthetic mixture of 11 CoA thioesters of interest was used to set up a chromatographic method. Using a porous organo-silica reversed phase column (100 × 2.1 mm, 1.5 µm), efficient separation of the analytes was achieved within 25 min, including the isobaric derivatives succinyl-CoA/methylmalonyl-CoA and methylsuccinyl-CoA/ethylmalonyl-CoA, respectively (Additional file 1: Fig. S1). As exception butyryl-CoA and isobutyryl-CoA co-eluted in all cases tested (data not shown). They could not be distinguished in the MS due to their identical mass either and were therefore regarded as one pool. The linear range for quantification covered 5–8 orders of magnitude, down to the picomolar level (Additional file 1: Fig. S2).

Next, we aimed to develop one common workflow, which was suitable to analyze real samples from the different microbes. Initial tests with *C. glutamicum* revealed that combined quenching and extraction was straightforward to handle experimentally and provided extracts of reproducible quality (data not shown), so that we used it as a starting point for development. Several practical challenges resulted from the nature of the different microbes and the used culture conditions and had to be addressed.

**Fig. 1:**
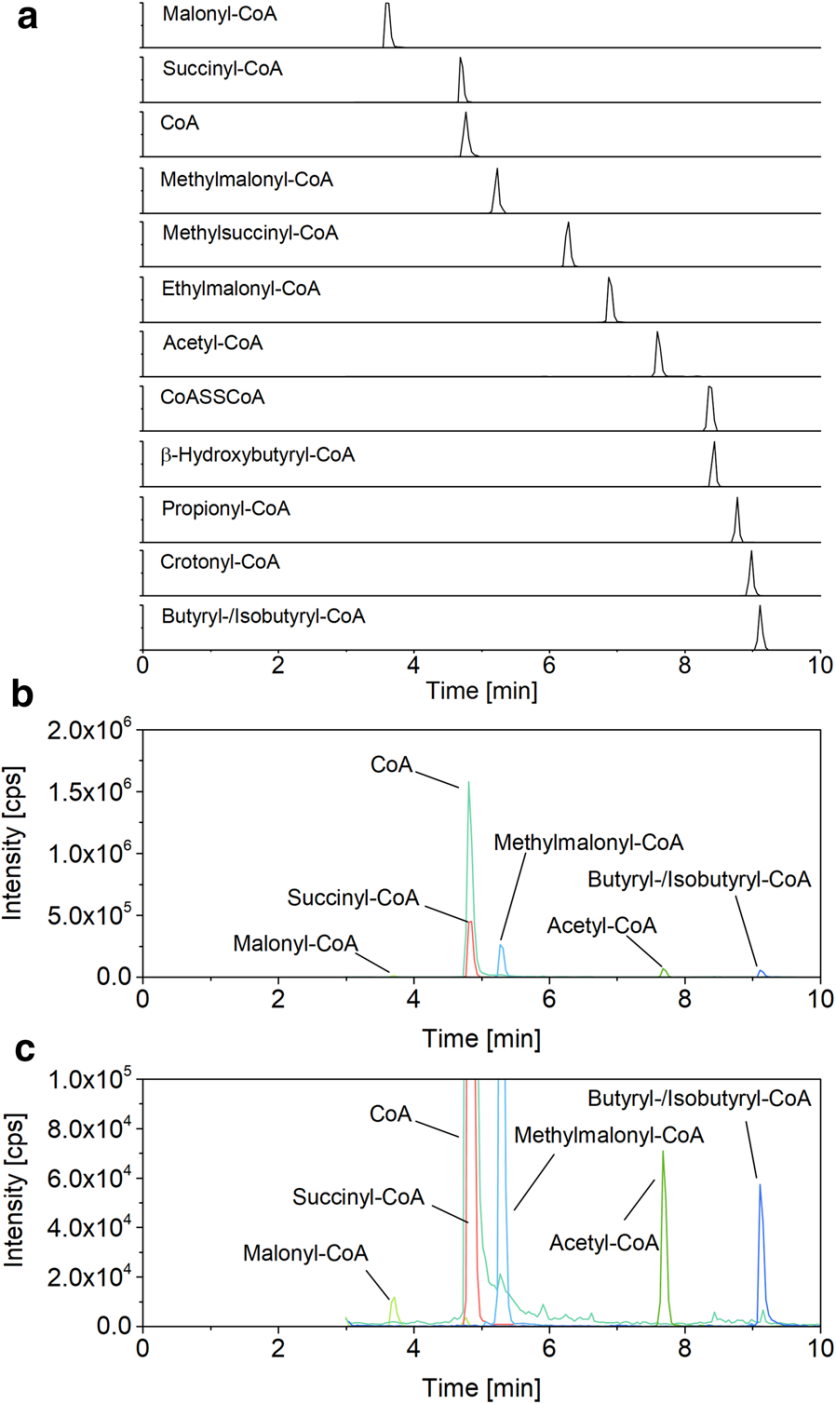
LC–MS/MS chromatogram of a synthetic CoA thioester standard sorted by the retention time of each analyte (**a**). LC–MS/MS chromatogram of *C. glutamicum* LYS-9 (**b**, **c**). Co-eluting CoA thioesters were distinguished by their different specific mass-to-charge ratio (*m/z*), except for butyryl- and isobutyryl-CoA. Analysis was performed on a triple quadrupole MS (AB Sciex QTRAP 6500^+^, AB Sciex Germany GmbH, Darmstadt, Germany) coupled to an HPLC system (Agilent Infinity 1290 System)

First, the chosen small column geometry and particle size turned out incompatible with certain samples. Over rather few injections (10–20), the column pressure increased from initially 250 to 1000 bar, which required extensive cleaning with water to regenerate the separation column. However, despite such efforts, we faced a rapid loss of separation efficiency. This was especially true for samples of *S. albus* and *C. glutamicum*, grown in media with elevated ionic strength. The use of a larger column (100 × 4.6 mm) and a twofold increased particle size of the separation material (3 µm) solved this issue so that more than 500 samples could be analyzed on the same column without pressure increase and loss in separation performance, independent of the microbe investigated. Due to the larger geometry, the eluent flow was increased to 600 µL min^−1^, which kept the analysis time at 25 min and provided a robust approach for the analytics. By far the best separation was achieved using a core–shell silica column instead of the porous silica column (Fig. 1). Due to superior properties of the core–shell material, the resulting peaks were narrower so that the gradient could be increased substantially. Altogether, this shortened the analysis time for all CoA esters to only 10 min (Fig. 1a). The analysis of cell extracts yielded clean chromatograms with high signal quality, even for low abundance thioesters (Fig. 1 b, c).

Second, small biomass amounts, typically chosen for sampling in metabolomics due to easier handling, were not suitable to precisely quantify all CoA thioesters present in vivo due to an extremely low abundance of some of them. As example, *S. albus* contained ultralow amounts of crotonyl-CoA, which yielded low quality signals near the threshold, when extracted from 0.6 mg biomass. Similar observations were made for the other microbes studied. An increase of the sample amount to 8 mg, however, allowed clean detection and quantification of all CoA thioesters to be expected from the genomic repertoire for each of the tested strains and conditions.

Third, the higher biomass amounts caused difficulties in dissolving lyophilized extracts, after freeze-drying them together with extracted cell fragments. The obtained solutions were too viscous, especially when sampling the filamentous actinomycete, to appropriately filter them prior to analysis. Due to this, we introduced a centrifugation step between extraction and lyophilization, which allowed a better handling, especially for *S. albus*. Additional tests for all strains revealed that after two washing cycles the cell pellets did not contain any significant residuals of the analytes of interest, which ensured complete extraction. In the following, ^13^C labeled cell extracts were prepared by growing each microbe on its corresponding [U-^13^C] substrate and conducting the established sample processing. The concentration of the ^13^C CoA thioesters was precisely quantified against synthetic standards so that the ^13^C extracts could then be used to quantify absolute concentrations.

Fourth, free CoA underwent dimerization to a certain degree during the sample processing. When analyzing the synthetic standard, approximately 15% ± 3% of free coenzyme A was observed as CoA-disulfide (CoA-S–S-CoA), eluting 3.5 min after the free monomer (Fig. 1, Additional file 1: Table S1). This phenomenon was also observed for cell extract samples.

### *C. glutamicum* reveals a small spectrum of CoA thioesters with methylmalonyl-CoA as the dominating metabolite

**Fig. 2:**
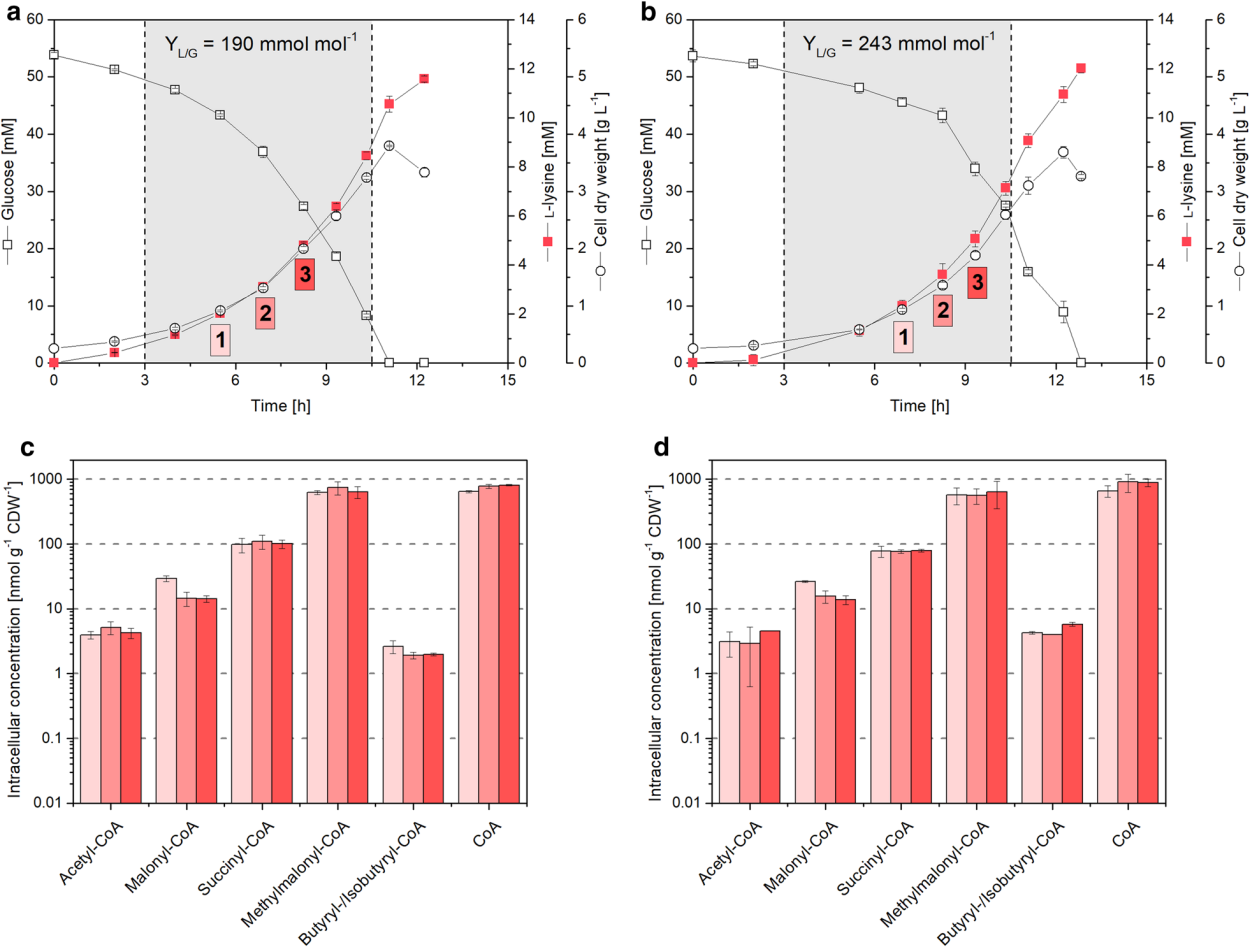
Absolute quantification of intracellular CoA thioesters in l-lysine producing *Corynebacterium glutamicum* LYS-9 (left) and LYS-9 ∆*sucCD* (right) during growth on glucose. The data comprise the time profile of glucose, l-lysine, and cell concentration (**a**, **b**) and absolute levels of intracellular CoA thioesters (**c**, **d**). The yield for l-lysine reflects the major production phase, indicated by the gray area, and analyzed for free CoA and CoA thioesters at three time points. The data for these time points are shown in subfigures **c** and **d**. n = 3

The l-lysine producing mutant *C. glutamicum* LYS-9 was analyzed during batch growth on glucose. It continuously accumulated l-lysine to a final titer of 10.5 mM at a yield of 190 mmol mol^−1^ (Fig. 2a). The specific growth rate remained constant over the whole cultivation (*µ *= 0.27 h^−1^). The cell interior of *C. glutamicum* LYS-9 contained five CoA thioesters: acetyl-CoA, malonyl-CoA, methylmalonyl-CoA, succinyl-CoA, and butyryl/isobutyryl-CoA. The esters differed almost 200-fold in abundance. Methylmalonyl-CoA exhibited the highest concentration (up to 750 nmol g^−1^), followed by succinyl-CoA (110 nmol g^−1^), malonyl-CoA (30 nmol g^−1^), acetyl-CoA (5 nmol g^−1^), and butyryl/isobutyryl-CoA (3 nmol g^−1^). Additionally, free coenzyme A was observed in significant amount (820 nmol g^−1^). The level of all CoA thioesters remained stable over time, except for malonyl-CoA, which decreased by approximately 50% in later cultivation stages (Fig. 2c, d). Incubated under the same conditions as its ancestor, *C. glutamicum* LYS-9 ∆*sucCD* (lacking succinyl-CoA synthetase) formed 12 mM l-lysine at an increased yield of 243 mmol mol^−1^, while growing at a specific growth rate of µ = 0.25 h^−1^ (Fig. 2b). The spectrum of intracellular CoA thioesters was almost unaffected in the TCA-cycle defective mutant, as compared to LYS-9. This was also true for succinyl-CoA, the substrate of the deleted enzyme. Its pool size was identical in both strains. Only butyryl/isobutyryl-CoA slightly differed (Student’s *t* test, *p *= 0.01).

### The actinomycete *S. albus* exhibits a rich set of CoA thioesters varying more than 10,000-fold in intracellular availability

**Fig. 3:**
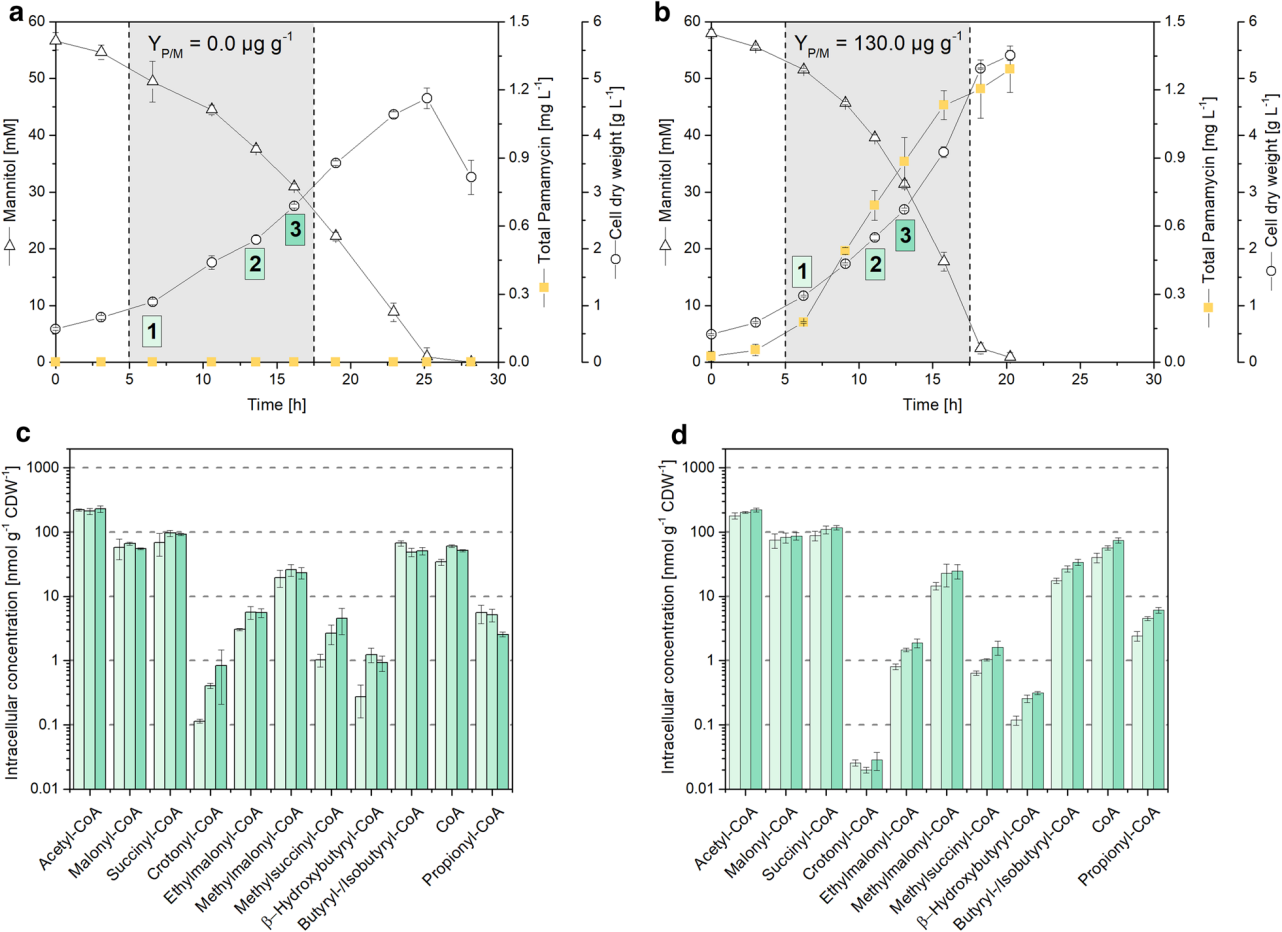
Absolute quantification of intracellular CoA thioesters in *Streptomyces albus* J1074 (left) and its pamamycin producing mutant J1074/R2 (right) during growth on mannitol. The data comprise the time profile of mannitol, total pamamycin and cell concentration (**a**, **b**), and absolute levels of intracellular CoA thioesters and free CoA (**c**, **d**). The yield for pamamycin reflects the major production phase, indicated by the gray area, and analyzed for CoA thioesters at three time points. n = 3

The wild type *S. albus* J1074 was grown on mannitol-based minimal medium (Fig. 3a). The substrate was consumed over a time of 25 h and cells reached a cell dry weight of 4.5 g L^−1^. CoA thioesters were sampled at three time points during the mid-growth phase. The actinomycete revealed a rich spectrum of eleven CoA thioesters with side chains of two, three, four and five carbons (Fig. 3c). Acetyl-CoA was most abundant (up to 230 nmol g^−1^), followed by succinyl-CoA, malonyl-CoA, and butyryl/isobutyryl-CoA. The other six thioesters partly exhibited much lower levels. Crotonyl-CoA and β-hydroxybutyryl-CoA were contained only in trace amounts down to 0.3 nmol g^−1^. Furthermore, *S. albus* contained free coenzyme A up to 60 nmol g^−1^. Along the cultivation, most pools (including those of high abundance) remained stable, but selected CoA thioesters changed to some extent. As example, the level of the carbon-five side-chain esters ethylmalonyl-CoA and methylsuccinyl-CoA increased over time.

The recombinant strain *S. albus* J1074/R2 produced 1.3 mg L^−1^ total pamamycin during growth on mannitol (Fig. 3b). The polyketide was produced from early on, accumulated in an exponential manner during the first hours and levelled off toward the end. The mutant revealed the same number of CoA thioesters as its ancestor *S. albus* J1074, but strongly differed in amount for some of them. As example, the level of crotonyl CoA was decreased up to more than ten-fold to 0.02 nmol g^−1^. In addition, the levels of β-hydroxybutyryl-CoA, ethylmalonyl-CoA, and methylsuccinyl-CoA were reduced up to five-fold (Fig. 3c, d). The other CoA thioesters, including pools of highest abundance (acetyl-CoA, malonyl-CoA, succinyl-CoA) appeared relatively unaffected by pamamycin production. Regarding the pamamycin spectrum, the strain synthetized various derivatives that differed in their mass, due to divergent side chains (Pam 579, Pam 593, Pam 607, Pam 621, Pam 635, Pam 649, Pam 663), which is known to be caused by the alternative incorporation of three-carbon malonyl-CoA, four-carbon methyl-malonyl-CoA, and five-carbon ethylmalonyl-CoA during biosynthesis. At the end of the process, the distribution was Pam 579 (1.5%), Pam 593 (5.6%), Pam 607 (40.5%), Pam 622 (48.1%), Pam 635 (3.9%), Pam 649 (0.3%) and Pam 663 (0.0%).

### Glucose-grown *P. putida* KT2440 shows a high abundance of free coenzyme A up to 1000-fold more than bound CoA thioesters

**Fig. 4:**
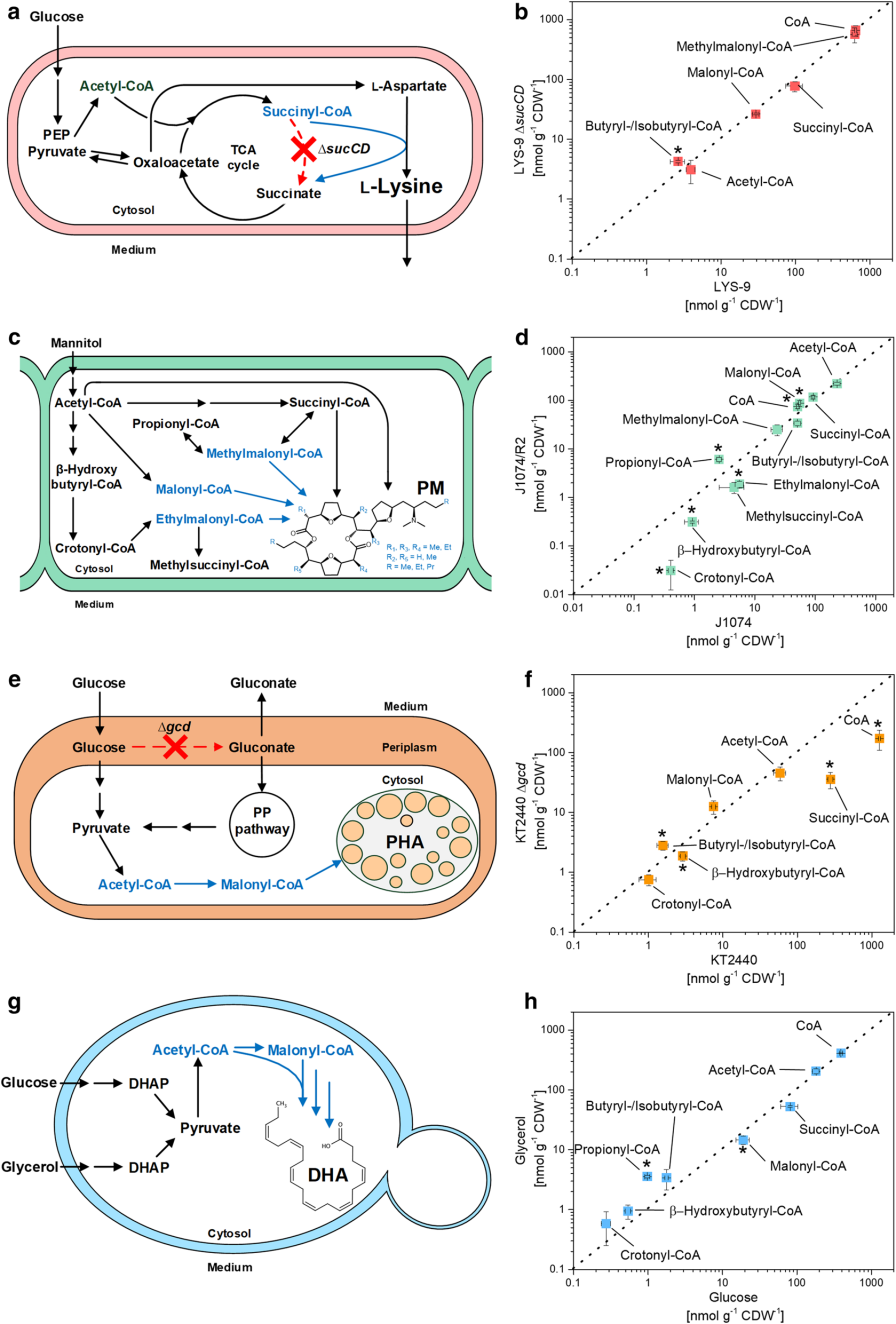
Impact of environmental and genetic perturbation on the spectrum of short-chain CoA thioesters and free coenzyme A in different microbes. The data show direct correlations in absolute CoA thioester levels between different strains of *Corynebacterium glutamicum* (**a**, **b**), *Streptomyces albus* (**c**, **d**), and *Pseudomonas putida* (**e**, **f**), and between glucose and glycerol grown *Yarrowia lipolytica* (**g**, **h**). The analysis comprised *C. glutamicum* LYS-9 and its succinyl-CoA synthetase deletion mutant LYS-9 ∆*sucCD*, which achieved a higher l-lysine yield, due to flux coupling of the l-lysine pathway with the disrupted TCA cycle (**a**, **b**) [15]. In comparison to the wild type *S. albus* J1074, the recombinant producer J1074/R2 formed the polyketide pamamycin from CoA thioester building blocks malonyl-CoA, methylmalonyl-CoA, ethylmalonyl-CoA, succinyl-CoA, and acetyl-CoA (**c**, **d**) [11]. In addition, the data comprise *Pseudomonas putida* KT2440 and its glucose dehydrogenase deficient mutant KT2440 ∆*gcd* (**e**, **f**) [13], and *Yarrowia lipolytica* Po1h::Af4 using glucose and glycerol as sole carbon source (**g**, **h**) [12]. The statistical significance for observed differences in CoA thioester levels (t-test *p *< 0.05) is marked by an asterisk. n = 3

When grown on glucose, *P. putida* KT2440 contained six intracellular CoA thioesters with two, three and four carbon side chains, respectively: acetyl-CoA, malonyl-CoA, succinyl-CoA, β-hydroxybutyryl-CoA, butyryl/isobutyryl-CoA, and crotonyl-CoA. The level of the CoA thioesters ranged from 280 nmol g^−1^ (succinyl-CoA) to 1 nmol g^−1^ (crotonyl-CoA). *P. putida* KT2440 contained a huge amount of free coenzyme A (1,260 nmol g^−1^), exceeding the sum of all thioester pools more than two-fold and the level of individual thioesters up to more than 1000-fold (Fig. 4f). The glucose dehydrogenase (*gcd*) deletion mutant KT2440 ∆*gcd*, grown under the same conditions, showed a five-fold decreased level for succinyl-CoA (p = 0.01) and β-hydroxybutyryl-CoA (0.6-fold, p = 0.01). The most obvious consequence of the *gcd* deletion was a dramatically decreased abundance of free coenzyme A (172 nmol g^−1^) (Fig. 4f).

### *Y. lipolytica* adapts the level of carbon three thioesters, when grown on glucose and glycerol

Acetyl-CoA, malonyl-CoA, butyryl/isobutyryl-CoA, β-hydroxybutyryl-CoA, crotonyl-CoA, and succinyl CoA were present, when the yeast was grown on glucose or on glycerol (Fig. 4h). The carbon source specifically affected the intracellular level of carbon-three CoA thioesters. Whereas malonyl-CoA was significantly increased on glucose (19 nmol g^−1^) as compared to glycerol (15 nmol g^−1^) (Student’s t-test, *p *= 0.04), propionyl-CoA was reduced more than threefold as compared glycerol-grown cells. The other CoA thioesters as well as free CoA showed similar concentrations on both substrates.

## Discussion

### The developed experimental workflow enables precise and reproducible quantification of CoA thioesters in gram-positive and gram-negative bacteria and eukaryotic yeast

Thioesters of coenzyme A play an important role in metabolism and participate in 5% of all enzymatic conversions [7]. However, only selected studies so far have managed to assess their presence in microbial cells using different protocols, specifically elaborated for the given question [8, 20, 22, 23]. In this work, we successfully adapted a workflow with integrated quenching and extraction using pre-cooled acetonitrile and formic acid, previously described for the methanol-utilizing bacterium *Methylobacterium extorquens* [21] to quantitatively extract intracellular CoA thioesters from *C. glutamicum, S. albus, P. putida,* and *Y. lipolytica*. The method precisely yielded absolute concentrations due to the use of internal ^13^C-standards. The addition of the standard during the initial extraction step allowed to compensate for potential concentration changes during sample processing, whereby all thioesters were found rather stable under the given conditions. Notably, the workflow should also deliver robust estimates of free CoA levels. This compound, due its reactive free thiol group, exhibited a certain degree of dimerization into the disulfide (and heterodimers might have been potentially formed with other reduced thiols such as glutathione and mycothiol). All these effects should be compensated by the internal standard (assuming that isotope effects in dimerization were negligible), underling its importance. One should, however, be aware of such side reactions and eventually explore them in more detail, when needed.

**Fig. 5:**
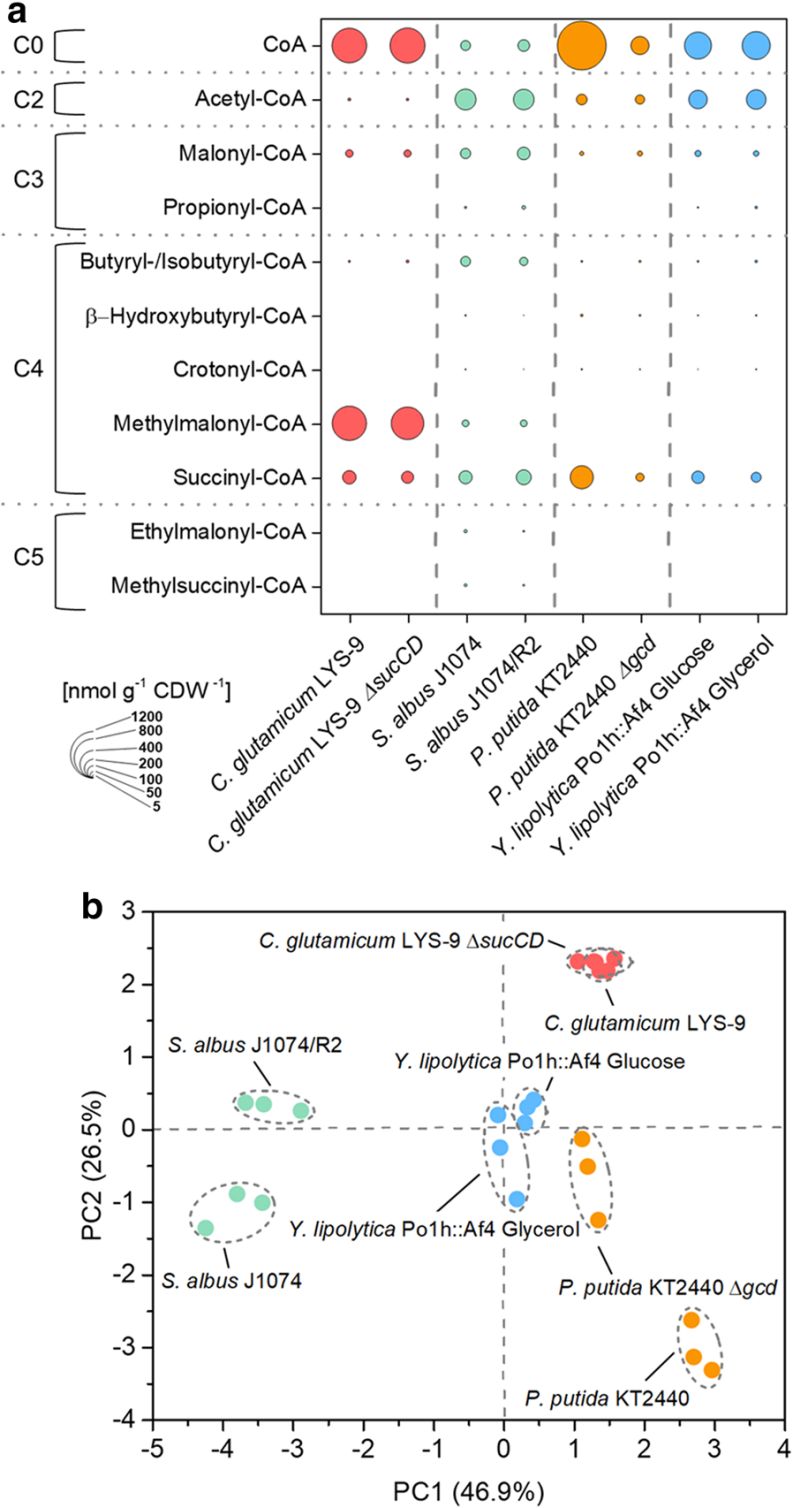
Spectra of short-chain CoA thioesters and free coenzyme A in different strains of *Corynebacterium glutamicum*, *Streptomyces albus*, *Pseudomonas putida*, and *Yarrowia lipolytica.* Absolute fingerprints of free CoA and carbon two to carbon five CoA thioesters for eight studied scenarios (**a**). Principal component analysis of the intracellular spectrum of CoA thioesters and free CoA of all studied scenarios (**b**). Detailed information on the studied strains is given in the legend to Fig. 3. n = 3

Following specific improvement in sampling, sample processing and analytics, we could separate, detect, and quantify CoA thioesters in cell extracts of all studied microbes down to the attomole level within only 10 min (Fig. 1, 3, and 4). Each microbe revealed a unique CoA thioester spectrum, regarding thioester number, type, and level (Fig. 5a). The method allowed to assess also low abundance CoA thioesters, such as crotonyl-CoA in recombinant *S. albus* (Fig. 4c, d). The wide linear range of 10^5^–10^8^ achieved for quantification (Additional file 1: Fig. S2) appeared crucial to cover the full spectrum of CoA thioesters, which differed more than 10,000-fold in intracellular concentration (Fig. 3d). Statistical analysis of the data, using principal component analysis, revealed that biological triplicates clustered closely for each experiment, independent of the studied strain (Fig. 5b). The concentrations, determined for single CoA esters, were in the same range as values previously observed in these and similar microbes [17–20]. The achieved high reproducibility appears valuable to identify even small phenotypic differences, particularly when considering the general difficulties to obtain precise metabolite and metabolome data [24]. The microbes selected in this study differed significantly in properties that potentially affect the suitability of experimental approaches in metabolomics: cell size and morphology, composition of the cell wall, and presence of specific cellular barriers, such as outer layers or compartmental membranes [25–27]. The fact that they all could be appropriately analyzed with the same workflow suggests a broad applicability of the method. The possibility to use of one common method for different microbes seems also interesting for automatized screening efforts, which more and more get into focus and benefit from standardized workflows [28].

In the following, the approach was applied to different industrial microbes to demonstrate its potential. As example, we could show that growth of *Y. lipolytica* on glycerol results in significantly enhanced levels of propionyl-CoA (Fig. 4h). This finding is interesting for the synthesis of odd-chain fatty acids in the yeast, which relies on propionyl-CoA availability and usually requires toxic propionate supplementation or massive strain engineering [29]. In contrast, glucose resulted in a higher amount of malonyl-CoA. In line, this substrate has proven more efficient than glycerol to derive PUFAs, built from this two CoA thioester, in *Y. lipolytica* [12].

*P. putida* KT2440 revealed a huge amount of free CoA, more than the other microbes (Fig. 5a), which might be involved in metabolic control, but requires more investigation. A disruption of the periplasmic oxidation route, the major pathway for glucose-breakdown [30] has been previously used to drive PHA synthesis in engineered *P. putida* [13]. As shown, the mutation did not significantly alter the availability of the PHA building blocks but affected the pools of free CoA and the TCA cycle intermediate succinyl-CoA, suggesting a broader impact on metabolism (Figs. 4e, f, and 5a). In addition, the CoA thioester analysis revealed interesting insights into the metabolism of *C. glutamicum* and *S. albus*, which are discussed below in more detail.

### The succinylase branch of l-lysine biosynthesis efficiently bridges the disrupted TCA cycle in succinate dehydrogenase deficient *C. glutamicum*

The amino acid l-lysine is an important industrial feed additive, largely produced with *C. glutamicum* [5]. The TCA cycle competes for carbon with l-lysine biosynthesis but is essential for the aerobic microbe and therefore cannot be eliminated [31]. Increased production, however, can be achieved by flux coupling of the TCA cycle to l-lysine biosynthesis [15]. Succinate dehydrogenase deficient strains cannot convert succinyl-CoA into succinate through the TCA cycle but use the succinylase branch of the l-lysine pathway instead, which results in significantly increased yield (Figs. 2a, b and 4b). One unanswered question so far related to the fact, how the genetic modification affected the availability of succinyl-CoA for l-lysine biosynthesis [15]. Here, we could show that a block of the TCA cycle at the level of succinate dehydrogenase did not affect the succinyl-CoA pool (Figs. 2 and 4b), and also not that of acetyl-CoA at the entry into the TCA cycle. The TCA cycle mutant and its parent strain exhibited an identical CoA thioester spectrum (Fig. 4b). This demonstrates that the three enzymes of the succinylase branch, succinyl-transferase (DapD), aminotransferase (DapC), and desuccinylase (DapE) [32] fully compensated for the disrupted TCA cycle. An insufficient capacity of this pathway would have otherwise presumably caused an accumulation of succinyl-CoA in the mutant. This finding displays a valuable insight into TCA cycle disrupted l-lysine hyper-producing strains. Future profiling of CoA thioesters seems also interesting for other *C. glutamicum* mutants, in which the l-lysine pathway [33], the TCA cycle [34] and pathways around the CoA thioester metabolism [35–37] have been engineered.

### The high abundance of methylmalonyl-CoA in *C. glutamicum* is promising towards heterologous production of complex polyketides

As shown, methylmalonyl-CoA was the dominating CoA thioester in *C. glutamicum* (Fig. 4b). From our data, we conclude that methylmalonyl-CoA is formed from succinyl-CoA by methylmalonyl-CoA mutase, eventually as response to TCA cycle activity. Propionyl-CoA, the potentially alternative source for methylmalonyl-CoA via propionyl-CoA carboxylase was proven absent so that this route can be excluded, matching with the fact that propionyl-CoA typically occurs as catabolic intermediate during the degradation of odd-chain fatty acids [38, 39] and branched-chain amino acids [40], not present here.

Methylmalonyl-CoA is a common extender substrate for the biosynthesis of complex polyketides by modular polyketide synthases [41]. The lack of this metabolite has been identified as a barrier to heterologous production of complex polyketides and extensive efforts have been made to install pathways to supply methylmalonyl-CoA as a building block [42, 43]. The discovered high abundance of methylmalonyl-CoA is therefore promising for future production of complex polyketides in *C. glutamicum*, which has been recently demonstrated via functional polyketide synthase expression and 6-methylsalicate biosynthesis in the microbe [36]. For future efforts, the 50-fold excess of methylmalonyl CoA over malonyl-CoA might display an interesting feature, because the relative availability of the two metabolites often impacts the final product structure due to promiscuous enzymes in polyketide synthase assembly lines [41]. Without doubt, the protocol for CoA thioester profiling developed in this work, appears useful for a broad characterization of precursor availability in polyketide producing *C. glutamicum* mutants.

### CoA thioester intermediates from the ethylmalonyl pathway are depleted in pamamycin-producing *S. albus* and indicate an impact of precursor availability on product formation

As shown, the CoA thioester spectrum significantly differed between the pamamycin-producing mutant of *S. albus* and the non-producing wildtype (Fig. 3). In particular, intermediates of the ethylmalonyl-CoA pathway [44] were decreased up to more than ten-fold in the producer: β-hydroxybutyryl-CoA, crotonyl-CoA, ethylmalonyl-CoA, and methylsuccinyl-CoA (Fig. 4d). The formation of pamamycin in the heterologous host obviously consumed more CoA thioester building blocks than were supplied from central metabolism. This could display a bottleneck towards higher titers and deserves further investigation in the future. It was interesting to note that introduction of the heterologous pamamycin pathway perturbed the ratio between malonyl-CoA, methylmalonyl-CoA, and ethylmalonyl-CoA. It was approximately 100:40:10 in the wild type and changed to 100:30:1 in the producer (Fig. 3c, d). The three building blocks compete for incorporation into pamamycin. Unusual polyketide synthases in the assembly line equally accept them as substrates, which leads to 16 pamamycin homologues that differ in their side chains at six positions [11, 45]. As shown from our data, the dramatically reduced availability of ethylmalonyl-CoA, together with the accumulation of malonyl-CoA, promoted the synthesis of smaller pamamycins. Indeed, 95.7% of all pamamycin derivatives observed (Pam 579, Pam 593, Pam 607, Pam 621) were light ones (had a lower mass), which could be formed without any contribution of ethylmalonyl-CoA. The pamamycins of higher mass (Pam 635, 649 and 663), which required one, two or even more ethylmalonyl-CoA units, became exceedingly rare, based on this effect. It would be interesting to further explore this link in other natural producers, which obviously differ in the spectrum of pamamycin homologues [46–48]. Metabolic engineering of CoA thioester supply appears promising to streamline pamamycin production towards selective derivatives, as proven valuable for other polyketides [18]. Likewise, a variation of bioprocess parameters appears promising to tailor the CoA ester spectrum [49].

## Materials and methods

### Microorganisms

Strains used in this study were obtained from previous work. This included *Streptomyces albus* J1074 and its pamamycin producing derivative J1074/R2 [11], the two l-lysine producing strains *Corynebacterium glutamicum* LYS-9 and LYS-9 *∆sucCD* [15], *Pseudomonas putida* KT2440 and its mutant KT2440 *∆gcd* [13], and the docosahexaenoic acid (DHA) producing recombinant yeast *Yarrowia lipolytica* Po1h::Af4 [12]. All strains were maintained as glycerol stocks at − 80 °C.

### Media

*Streptomyces albus* was kept on mannitol-soy flour (MS) agar containing per liter: 20 g mannitol, 20 g soy flour (Schoenenberger Hensel, Magstadt, Germany), and 20 g agar (Becton–Dickinson, Heidelberg, Germany) [49]. Liquid pre-cultures of *S. albus* were grown in LB broth (20 g L^−1^, Sigma-Aldrich, Darmstadt, Germany) and main cultures were grown in minimal medium, which contained per liter: 10 g mannitol, 200 mM potassium phosphate buffer (pH 7.8), 15 g (NH_4_)_2_SO_4_, 1 g NaCl, 550 mg MgCl_2_*7H_2_O, 200 mg CaCl_2_, 30 mg 3,4-dihydroxybenzoic acid, 20 mg FeSO_4_, 2 mg FeCl_3_*6H_2_O, 2 mg MnSO_4_*H_2_O, 0.5 mg ZnSO_4_*H_2_O, 0.2 mg CuCl_2_*2H_2_O, 0.2 mg Na_2_B_4_O_7_*10H_2_O, 0.1 mg (NH_4_)_6_Mo_7_O_24_*4H_2_O, 1 mg nicotinamide, 1 mg riboflavin, 0.5 mg thiamine hydrochloride, 0.5 mg pyridoxine hydrochloride, 0.2 mg biotin, and 0.1 mg p-aminobenzoic acid. In addition, liquid media were amended with 30 g L^−1^ glass beads (soda-lime glass, 5 mm, Sigma-Aldrich) to avoid cell agglomeration.

*Corynebacterium glutamicum* was kept on BHI agar (37 g L^−1^ BHI, 20 g L^−1^ agar, Becton–Dickinson). Pre-cultures and main cultures of *C. glutamicum* were grown on complex BHI medium and minimal glucose medium, respectively, as described previously [3].

*Pseudomonas putida* was kept on BHI agar (37 g L^−1^ BHI, 20 g L^−1^ agar, Becton–Dickinson). The mineral M9 medium, used for all liquid cultures, contained per liter: 20 g glucose, 12.8 g Na_2_HPO_4_*7H_2_O, 3 g KH_2_O_4_, 1 g NH_4_Cl, 0.5 g NaCl, 0.25 g MgSO_4_*7H_2_O, 6 mg FeSO_4_*7H_2_O, 2.7 mg CaCO_3_, 2.0 mg ZnSO_4_*H_2_O, 1.2 mg MnSO_4_*H_2_O, 0.4 mg CoSO_4_*7H_2_O, 0.3 mg CuSO_4_*5H_2_O, and 0.1 mg H_3_BO_3_ [13].

*Yarrowia lipolytica* was incubated on YNB-N5000 agar, which contained per liter: 10 g glucose, 5 g (NH_4_)_2_SO_4_, 1.7 g YNB (yeast nitrogen base w/o amino acids and ammonium sulfate, Sigma-Aldrich), and 20 g agar. All liquid cultures of the yeast were conducted in minimal medium, containing per liter: 10 g glycerol or 10 g glucose, 200 mM 2-(*N*-morpholino)ethanesulfonic acid (MES, pH 6.8), 5 g (NH_4_)2SO_4_, and 1.7 g YNB.

### Cultivation in shake flasks

Liquid cultures were incubated in baffled shake flasks (500 mL, 10% filling volume) on an orbital shaker (Multitron, Infors AG, Bottmingen, Switzerland, 5 cm shaking diameter, 230 rpm, 75% relative humidity), whereby the temperature was adjusted individually (30 °C for *P. putida* and *C. glutamicum*; 28 °C for *S. albus* and *Y. lipolytica*). For each strain, a specific protocol for inoculation and pre-culturing was used to obtain reproducibly growing main cultures. *S. albus* was incubated on MS agar at 28 °C for 3 days until sporulation occurred. Spores of a single colony were collected to inoculate the pre-culture, which was incubated overnight in LB medium. Afterwards, cells were collected (5000×*g*, 25 °C, 6 min), resuspended in main culture medium, and used to inoculate the main culture. *C. glutamicum* was grown overnight on BHI agar at 30 °C. A single colony was used to inoculate an overnight pre-culture, which was then collected (5,000 x*g*, 25 °C, 6 min), resuspended in main culture medium, and used to inoculate the main culture. *P. putida* was grown overnight on M9 agar (30 °C). A single colony served as inoculum for the pre-culture which was then grown overnight, harvested (5,000 x*g*, 25 °C, 6 min), resuspended in main culture medium, and used to inoculate the main culture. *Y. lipolytica* was grown overnight on YNB-N5000 agar at 28 °C. A single colony was used to inoculate the pre-culture, which was incubated overnight, harvested (5000×*g*, 25 °C, 6 min), resuspended in main culture medium and then served as inoculum for the main culture. All growth experiments were conducted as biological triplicate.

### Determination of cell concentration

All investigated microbes were analyzed for their cell dry weight. Cells of *S. albus* were vacuum-filtered using a nitrocellulose filter (0.2 µM, Sartorius, Göttingen, Germany), washed twice with 15 mL deionized water, and gravimetrically analyzed using a moisture analyzer (HB43-S, Mettler-Toledo, Columbus, USA). The parallel measurement of the cell concentration as optical density at 600 nm (OD_600_) resulted in a correlation factor of CDW (g L^−1^) = 0.62 × OD_600_. The cell dry weight of *C. glutamicum* was inferred from the optical density measurement at 660 nm as previously described [3]. The cell dry weight of *P. putida* and *Y. lipolytica* was measured as follows. Cells were collected (15,000×*g*, 4 °C, 10 min), washed twice with 15 mL deionized water, and freeze-dried. Afterwards, the dry biomass was gravimetrically determined.

### Quantification of substrates

Mannitol and glucose were quantified by HPLC (1260 Infinity Series, Agilent, Darmstadt, Germany) using a Metacarb 87C column (300 × 7.8 mm, Agilent), a Metacarb 87C guard column (50 × 7.8 mm, Agilent), a desalting column (Microguard Deashing Cartridge, Bio-Rad, Munich, Germany), and demineralized water as mobile phase (85 °C, 0.6 mL min^−1^). Refraction index measurement was used for detection, and external standards were used for quantification [2, 3].

### Extraction and quantification of pamamycins

Prior to analysis, pamamycins were extracted from *S. albus* culture broth. For this purpose, 200 µL broth was mixed with 200 µL acetone and incubated for 15 min (1,000 rpm, room temperature, Thermomixer F1.5, Eppendorf, Wesseling, Germany). Afterwards, 200 µL ethyl acetate was added, and the mixture was incubated for further 15 min. The organic phase was collected by centrifugation (20,000×*g*, 5 min, room temperature). Subsequently, the solvent mixture was evaporated under nitrogen. The obtained extract was dissolved in methanol and clarified from debris (20,000 x*g*, 5 min, 4 °C). Afterwards, the different pamamycin derivatives were analyzed using LC–ESI–MS/MS (QTRAP 6500^+^, AB Sciex, Darmstadt, Germany) coupled to an HPLC system (Agilent Infinity 1290 System). In short, the analytes were separated on a C18 column (Vision HT C18 HighLoad, 100 mm × 2 mm, 1.5 µm, Dr. Maisch, Ammerbuch-Entringen, Germany) at 45 °C and a flow rate of 300 µL min^−1^ (8 mM ammonium formate in 92% acetonitrile). Detection was carried out in positive selected ion monitoring (SIM) mode, using the [M + H]^+^ ion for each pamamycin derivative.

### Quantification of l-lysine

The amino acid l-lysine was quantified using HPLC with pre-column derivatization and fluorescence detection as described before [50]. For quantification, α-aminobutyric acid was used as internal standard [2].

### Extraction of intracellular CoA thioesters

A broth sample (approximately 8 mg CDW) was collected and immediately transferred into a pre-cooled extraction and quenching buffer (95% acetonitrile, 25 mM formic acid, -20 °C) [21]. The volume ratio was 1:4. The obtained solution was thoroughly mixed while cooled on ice for 10 min, and then clarified from debris (15,000×*g*, 4 °C, 10 min). The obtained supernatant was mixed with 10 mL super cooled deionized water (− 2 °C). The cell pellet was twice washed with 8 mL super cooled deionized water. Afterwards, all supernatants were combined, frozen with liquid nitrogen, freeze-dried, and then re-dissolved in 500 µL pre-cooled resuspension buffer (25 mM ammonium formate, pH 3.0, 2% MeOH, 4 °C) [51]. The buffered extract was filtered (Ultrafree-MC 0.22 µm, Merck, Millipore, Germany) prior to analysis.

### Quantification of CoA thioesters using LC–ESI–MS/MS

The analysis of CoA thioesters was performed on a triple quadrupole MS (QTRAP 6500^+^, AB Sciex, Darmstadt, Germany) coupled to an HPLC system (Agilent Infinity 1290 System). Generally, the injection volume was 10 µL. Separation of the analytes of interest was conducted on a porous reversed phase column (Gemini C18, 100 mm × 4.6 mm, 3 µm, 110 Å, Phenomenex, Aschaffenburg, Germany) at 40 °C using a gradient of formic acid (50 mM, adjusted to pH 8.1 with ammonium hydroxide 25% in H_2_O, eluent A) and methanol (eluent B) at a flow rate of 600 µL min^−1^. The fraction of eluent B was as follows: 0–12 min, 0–15% B; 12–16 min, 15–100% B; 16–18 min, 100% B; 18–20 min, 100–0% B; 20–25 min, 0% B. Initial tests were further done, using a smaller column geometry and pore size (Gemini C18, 100 mm × 2.1 mm, 1.5 µm, 110 Å, Phenomenex), using the same gradient, but a reduced flow rate of 300 µL min^−1^. In addition (and finally used in the optimized workflow), a core–shell reversed phase column (Kinetex XB-C18, 100 × 2.1 mm, 2.6 µm, 100 Å, Phenomenex) was applied at 40 °C, using a gradient of formic acid (50 mM, adjusted to pH 8.1 with ammonium hydroxide 25% in H_2_O, eluent A) and methanol (eluent B) at a flow rate of 300 µL min^−1^. The fraction of eluent B was as follows: 0–7 min, 0–10% B; 7–10 min, 10–100% B; 10–11 min, 100% B; 11–12 min, 100–0% B; 12–15 min, 0% B. During the first 3 min of the analysis, the outflow from the chromatographic column was discharged to minimize the entry of salts from samples into the mass spectrometer. The individual CoA thioesters were detected using multiple reaction monitoring (MRM), involving the corresponding parent ion and its respective daughter ion (Additional file 1: Table S1). Further instrument settings were as follows: curtain gas, 35 psi; collision gas flowrate, medium; ion spray voltage, 4.5 kV; temperature, 400 °C; ion source gas, 60 psi; and entrance potential, 10 V. The declustering potential, the collision energy and the collision cell exit potential were optimized individually for each CoA thioester using synthetic standards. Acetyl-CoA, propionyl-CoA, succinyl-CoA, methylmalonyl-CoA, and free CoA were purchased (Sigma-Aldrich), whereas malonyl-CoA, β-hydroxybutyryl-CoA, butyryl-CoA, isobutyryl-CoA, crotonyl-CoA, methylsuccinyl-CoA and ethylmalonyl-CoA were chemo-enzymatically synthesized as previously described [7].

### Absolute quantification of CoA thioesters using ^13^C-labeled extracts

Absolute quantification of CoA thioesters was conducted using the MIRACLE approach [52]. For this purpose, ^13^C-labeled cell extracts were used as internal standard, whereby an individual standard was produced for each microbe. For this purpose, the different organisms were grown on ^13^C-enriched substrates, i.e. the naturally labeled carbon source was replaced by an equimolar amount of the [U-^13^C] enriched isomer: 99% [^13^C_6_] d-mannitol (*S. albus*), 99% [^13^C_6_] d-glucose (*P. putida* and *C. glutamicum*), and 99% [^13^C_3_] d-glycerol (*Y. lipolytica*). The ^13^C tracer substrates were obtained from Cambridge Isotope Laboratories (Tewksbury, MA, USA). For each microbe, the second pre-culture and the main culture was conducted in the ^13^C-enriched minimal medium. In each case, the inoculum size was below 1% of the later harvested cell concentration to finally provide fully ^13^C enriched cell extracts as standard and exclude potential interference [27]. The culture broth from the main ^13^C culture was extracted during the late exponential growth phase, using the extraction protocol described above. After freeze-drying and re-suspension, the ^13^C extract was stored as aliquots at − 80 °C. The level of the individual CoA thioesters in each ^13^C extract was precisely quantified using the synthetic standards and corresponding instrument settings (Additional file 1: Tables S1 and S2). During later analysis, an appropriate volume of ^13^C extract (of the respective microbe) was thawn on ice, and then simultaneously added with the sample into the quenching solution. This protocol allowed to to infer absolute metabolite levels and to take any eventual changes during sample processing into account.

### Principal component analysis

Principle component analysis (PCA) was performed using the ClustVis web tool [53].

## Supplementary information

**Additional file 1: Figure S1.** LC-MS chromatogram of a synthetic CoA thioester standard using a porous organo-silica reversed phase column (100 × 2.1 mm, 1.5 µm) for the chromatographic separation. Co-eluting analytes were distinguished by a different specific mass-to-charge ratio (*m/z*). **Figure S2.** Calibration curves for different CoA thioesters using LC-MS/MS analysis. **Table S1.** Instrumental settings for LC-MS/MS analysis of CoA thioesters. The declustering potential (DP), the collision energy (CE) and the cell exit potential (CXP) were individually tuned for each CoA thioester. The parent ion reflects the positive proton adduct [M+H]^+^, except for the CoA homodimer (CoA-S-S-CoA), where the parent ion was [M+2H]^2+^. In each case, the daughter ion reflects the positive proton adduct after neutral loss of 507 (*m/z*). **Table S2.** Instrumental settings for LC-MS/MS analysis of fully ^13^C-labeled CoA thioesters used as internal standard for absolute CoA thioesters quantification. The respective mass of the fully labeled parent ion was determined by adding the number of carbon atoms to the monoisotopic mass of the non-labelled parent ion (Table S1). The mass of each daughter ion was then calculated by subtraction of *m/z* 517 form this value, considering the neutral loss of a fragment with ten ^13^C atoms.

## Data Availability

The dataset(s) supporting the conclusions of this article are all included within the article.
