# Supplementary material for: A common approach for absolute quantification of short chain CoA thioesters in prokaryotic and eukaryotic microbes
Source: Microb Cell Fact. 2020 Aug 10;19:160. doi: 10.1186/s12934-020-01413-1 (PMC7418318; doi:10.1186/s12934-020-01413-1)
Supplement: Supplementary file 1 — Additional file 1: Figure S1. LC-MS chromatogram of a synthetic CoA thioester standard using a porous organo-silica reversed phase column (100 × 2.1 mm, 1.5 µm) for the chromatographic separation. Co-eluting analytes were distinguished by a different specific mass-to-charge ratio (m/z). Figure S2. Calibration curves for different CoA thioesters using LC-MS/MS analysis. Table S1. Instrumental settings for LC-MS/MS analysis of CoA thioesters. The declustering potential (DP), the collision energy (CE) and the cell exit potential (CXP) were individually tuned for each CoA thioester. The parent ion reflects the positive proton adduct [M+H]+, except for the CoA homodimer (CoA-S-S-CoA), where the parent ion was [M+2H]2+. In each case, the daughter ion reflects the positive proton adduct after neutral loss of 507 (m/z). Table S2. Instrumental settings for LC-MS/MS analysis of fully 13C-labeled CoA thioesters used as internal standard for absolute CoA thioesters quantification. The respective mass of the fully labeled parent ion was determined by adding the number of carbon atoms to the monoisotopic mass of the non-labelled parent ion (Table S1). The mass of each daughter ion was then calculated by subtraction of m/z 517 form this value, considering the neutral loss of a fragment with ten 13C atoms. [file 12934_2020_1413_MOESM1_ESM.pdf]

Additional file 1 for

A common approach for absolute quantification of short chain CoA thioesters in prokaryotic and eukaryotic microbes

Lars Gläser[1], Martin Kuhl[1], Sofija Jovanovic[1], Michel Fritz[1], Bastian Vögeli[2], Tobias J. Erb[2], Judith Becker[1] and Christoph Wittmann[1#]

[1]Institute of Systems Biotechnology, Saarland University, Saarbrücken, Germany

[2]Max Planck Institute for Terrestrial Microbiology, Marburg, Germany

[#] Phone/FAX: +49 681 302 71970/71972, e-mail: christoph.wittmann@uni-saarland.de

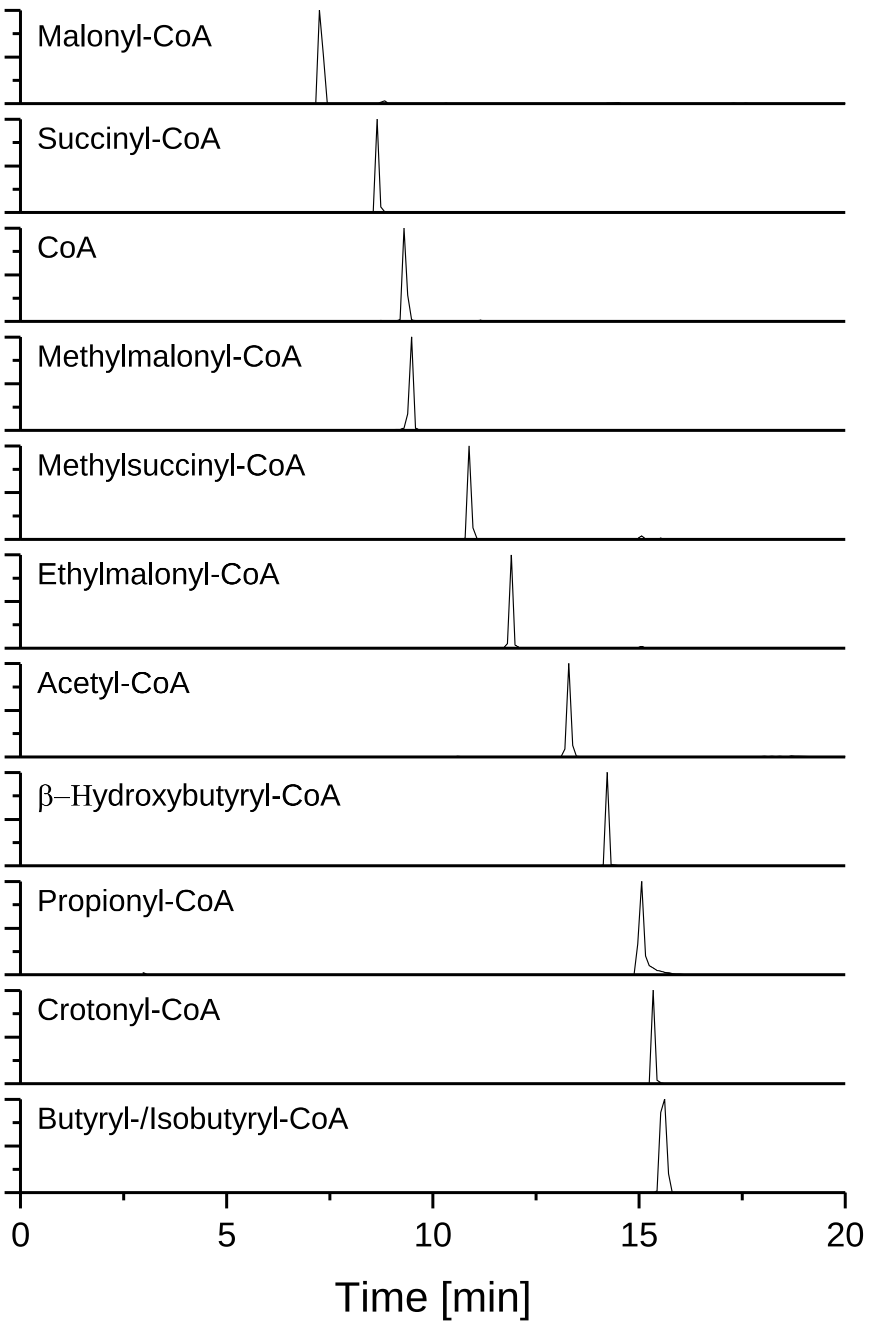

Figure S1: LC-MS chromatogram of a synthetic CoA thioester standard using a porous organo-silica reversed phase column (100 × 2.1 mm, 1.5 µm) for the chromatographic separation. Co-eluting analytes were distinguished by a different specific mass-to-charge ratio (*m/z*).

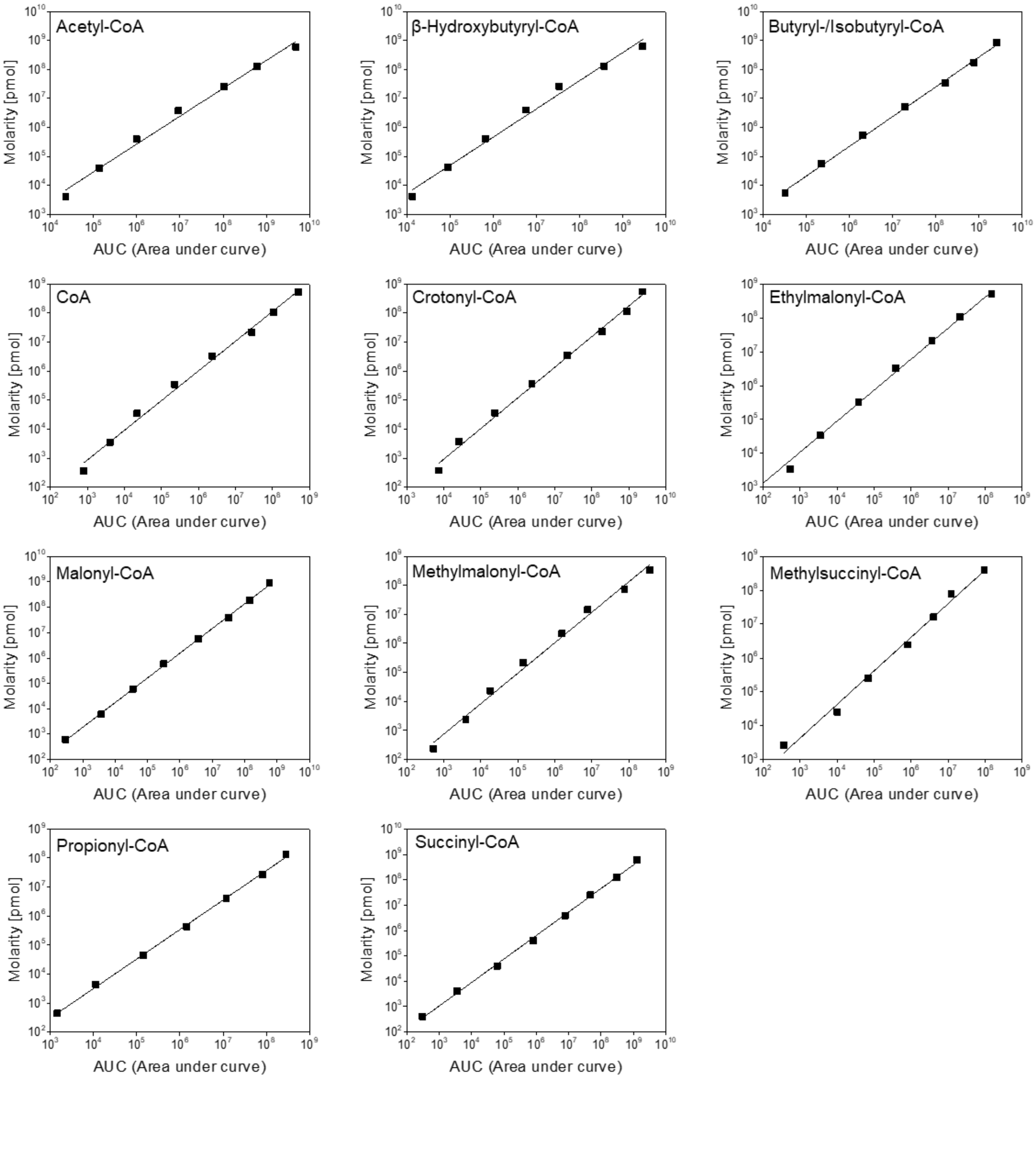

Figure S2: Calibration curves for different CoA thioesters using LC-MS/MS analysis.

Table S1: Instrumental settings for LC-MS/MS analysis of CoA thioesters. The declustering potential (DP), the collision energy (CE) and the cell exit potential (CXP) were individually tuned for each CoA thioester. The parent ion reflects the positive proton adduct $[M+H]^{+}$, except for the CoA homodimer (CoA-S-S-CoA), where the parent ion was $[M+2H]^{2+}$ In each case, the daughter ion reflects the positive proton adduct after neutral loss of 507 (*m/z*).

**Analyte**	**Parent ion (*m/z*)**	**Daughter ion (*m/z*)**	**DP [V]**	**CE [V]**	**CXP [V]**
CoA-S-S-CoA	767.4	136.0	80.00	47.00	11.00
CoA	768.5	261.0	80.00	47.00	11.00
Acetyl-CoA	810.2	303.0	154.57	38.87	17.01
Malonyl-CoA	854.0	346.6	46.50	43.84	21.75
Propionyl-CoA	824.6	317.1	80.00	47.00	11.00
Butyryl-/Isobutyryl-CoA	838.1	331.1	128.22	45.50	22.16
β-Hydroxybutyryl-CoA	854.1	347.1	142.36	39.95	10.73
Crotonyl-CoA	836.1	329.1	143.00	44.00	20.37
Methylmalonyl-CoA	868.0	361.1	90.06	42.50	11.87
Succinyl-CoA	868.1	361.1	15.96	46.78	21.68
Ethylmalonyl-CoA	882.1	375.1	177.58	37.80	25.30
Methylsuccinyl-CoA	882.0	375.1	131.79	50.40	23.73

Table S2: Instrumental settings for LC-MS/MS analysis of fully ^{13}C-labeled CoA thioesters used as internal standard for absolute CoA thioesters quantification. The respective mass of the fully labeled parent ion was determined by adding the number of carbon atoms to the monoisotopic mass of the non-labelled parent ion (Table S1). The mass of each daughter ion was then calculated by subtraction of *m/z* 517 form this value, considering the neutral loss of a fragment with ten ^{13}C atoms.

**Analyte**	**Parent ion (*m/z*)**	**Daughter ion (*m/z*)**	**DP [V]**	**CE [V]**	**CXP [V]**
CoA-S-S-CoA	788.1	141.1	80.00	47.00	11.00
CoA	789.5	272.5	80.00	47.00	11.00
Acetyl-CoA	833.2	316.2	154.57	38.87	17.01
Malonyl-CoA	878.0	361.0	46.50	43.84	21.75
Propionyl-CoA	848.6	331.6	80.00	47.00	11.00
Butyryl-/Isobutyryl-CoA	863.1	346.1	128.22	45.50	22.16
β-Hydroxybutyryl-CoA	879.1	362.1	142.36	39.95	10.73
Crotonyl-CoA	861.1	344.1	143.00	44.00	20.37
Methylmalonyl-CoA	893.0	376.0	90.06	42.50	11.87
Succinyl-CoA	893.1	376.1	15.96	46.78	21.68
Ethylmalonyl-CoA	908.1	391.1	177.58	37.80	25.30
Methylsuccinyl-CoA	908.0	391.0	131.79	50.40	23.73
